# Imerslund-Gräsbeck syndrome: a comprehensive review of reported cases

**DOI:** 10.1186/s13023-023-02889-x

**Published:** 2023-09-14

**Authors:** Sandra D.K. Kingma, Julie Neven, An Bael, Marije E.C. Meuwissen, Machiel van den Akker

**Affiliations:** 1 Centre for Metabolic Diseases, University Hospital Antwerp, University of Antwerp, Drie Eikenstraat 655, Edegem, Antwerp, 2650 Belgium; 2 Department of Pediatrics, University Hospital Antwerp, University of Antwerp, Drie Eikenstraat 655, Edegem, 2650 Belgium; 3 Faculty of medicine and health sciences, University of Antwerp, Antwerp, Belgium; 4 Department of Pediatric Nephrology, ZNA Queen Paola Children’s Hospital, Lindendreef 1, Antwerp, 2020 Belgium; 5 Center of Medical Genetics, University Hospital Antwerp, Drie Eikenstraat 655, Edegem, 2650 Belgium; 6 Department of Pediatrics, ZNA Queen Paola Children’s Hospital, Lindendreef 1, Antwerp, 2020 Belgium; 7 Pediatric Hematology and Oncology, Department of Pediatrics, University Hospital Antwerp, Drie Eikenstraat 655, Edegem, Antwerp, 2650 Belgium

**Keywords:** Imerslund-Gräsbeck syndrome, Vitamin B12 deficiency, Cobalamin deficiency, Vitamin B12 malabsorption

## Abstract

Imerslund-Gräsbeck syndrome (IGS) is a rare autosomal recessive disorder characterized by vitamin B12 malabsorption. Most patients present with non-specific symptoms attributed to vitamin B12 deficiency, and proteinuria. Patients may if untreated, develop severe neurocognitive manifestations. If recognized and treated with sufficient doses of vitamin B12, patients recover completely. We provide, for the first time, an overview of all previously reported cases of IGS. In addition, we provide a complete review of IGS and describe two new patients.

**Supplementary Information:**

The online version contains supplementary material available at 10.1186/s13023-023-02889-x.

## Background

Imerslund-Gräsbeck syndrome (IGS, OMIM 261,100/618,882) is an autosomal recessive disorder characterized by a vitamin B12 (vitB12) deficiency, caused by a selective malabsorption of vitB12, with normal secretion of intrinsic factor (IF) and hydrochloric acid into the stomach [1]. Before the diagnosis of vitB12 deficiency, patients have non-specific symptoms such as failure to thrive, paleness, fatigue, recurring viral infections and mild neurological symptoms. Laboratory examination often reveals macrocytic anemia with or without proteinuria [1]. IGS was first described in 1960 by Imerslund [2] and Gräsbeck et al. [3]. The reports included ten and two patients, respectively, although similar cases had been published since the early 19th century [4]. IGS is caused by mutations in the gene *CUBN* [Genbank NG_008967] encoding cubilin or *AMN* [Genbank NG_008276] encoding amnionless [5]. Homozygous or compound heterozygous mutations in either *CUBN* or *AMN* lead to IGS [1]. Cubilin and amnionless are the two subunits of the vitB12-IF receptor of the ileal mucosa, known as cubam. It is considered essential for intestinal absorption of vitB12, as well as for renal protein reabsorption. Most IGS patients are treated with intra-muscular vitB12, which most frequently corrects the clinical phenotype [1].

IGS is a rare disorder with an estimated prevalence to be less than 6 per 1,000,000 ^1^. We report on two siblings with IGS, which prompted us to provide an overview of all previously reported cases and a review of IGS.

## New cases

The patient was a 4-year-old girl of Turkish parents. Medical history was uneventful. Parents were consanguinous, all grandparents were interrelated. The brother of our patient had congenital adrenal deficiency due to a homozygous pathogenic deletion of exon 1–3 of *CYP21A2* [Genbank NM_007941] mutation. Futhermore, a sister who was born with hydrops fetalis died three days after birth.

Our patient presented at our outpatient clinic with three days of fever and coughing. Clinical examination and laboratory analysis suggested a viral infection. Unexpectedly, a slight decrease in hemoglobin was observed, in addition to high mean corpuscular hemoglobin, high mean corpuscular volume, thrombocytopenia, and leukopenia (Table 1). Furthermore, a high lactate dehydrogenase (LDH) and low vitB12 were observed. The peripheral blood smear showed several abnormalities and abnormal cells, with tear drop cells as the most apparent. Teardrop cells are described in a wide range of diseases that are associated with bone marrow fibrosis, such as myelodysplastic syndromes, leukemia, metabolic disorders, hemolytic anemia, iron deficits, thalassemia, but have also been described in macrocytic anemia [6]. There was no evidence of hemolytic anemia, ferritin was in the normal range and hemoglobin electrophoresis showed no evidence of a hemoglobinopathy such as thalassemia.

**Table 1:** Laboratory examination of patient 1

	Patient	Unit	Normal range
Hemoglobin	10.7	g/dL	11.0-13.6
Hematocrit	0.28	L/L	0.32–0.40
Erythrocyte count	2.75	× 10e12/L	4–5
Mean corpuscular hemoglobin	39	pg	26–30
Mean corpuscular volume	103	fL	76–87
Red blood cell distribution width	30.3	%	12.3–14.3
Reticulocyte count	66	× 10e9/L	43–83
Thrombocyte count	91	× 10e9/L	193–489
Leukocyte count	3.6	× 10e9/L	5–12
*Peripheral smear*			
Neutrophil count	0.63	× 10e9/L	2.1–8.9
Eosinophil count	0.05	× 10e9/L	0.0-0.3
Basophil count	0	× 10e9/L	0.0-0.5
Lymphocyte count	2.89	× 10e9/L	1.2–4.5
Monocyte count	0.02	× 10e9/L	0.5–1.1
Erythroblast count	5	/100WBC	0
Blast count	0	%	0
*Morphology red blood cells*			
Poikilocytosis	++		
Polychromasia	++		
Anisocytosis	+++		
Microcytosis	+		
Macrocytosis	+		
Teardrop cells	+++		
Acanthocytes	+		
Stomatocytes	+		
Basophilic inclusions	+		
Pappenheimer bodies	+		
Lactate dehydrogenase	5394	U/L	425–975
Uric acid	2.9	mg/dL	2.2–4.7
Creatinine	< 0.15	mg/dL	0.1–0.7
Folic acid	17	ng/mL	4–27
Vitamin B12	< 100	ng/L	313–1410
Ceruloplasmin	0.25–0.45	g/L	0.3
Ferritin	60	µg/L	6-137
Homocystein	44	µmol/L	< 15
Bilirubin			

Because high LDH and pancytopenia may be signs of myelodysplastic syndrome and leukemia, the bone marrow was examined. The bone marrow contained an increase in erythroblasts, but no other abnormalities.

Urinalysis revealed proteinuria (1.04 g/L, normal range: <0.12). Due to the combination of megaloblastic anemia, vitB12 deficiency and proteinuria, screening for abnormalities in the *AMN* and *CUBN* gene was performed. A homozygous variant in *AMN*, c.208-2 A > G, was identified. The variant was previously reported in the literature and considered pathogenic.

Oral supplementation with vitB12 was initiated, correcting the vitB12 deficiency, anemia, and related symptoms.

The brother of patient 1 was tested for IGS and the same homozygous pathogenic variant c.208-2 A > G mutation in *AMN* was detected at 8 months of age, in addition to the homozygous partial deletion of *CYP21A2*. He had an adrenal crisis at the age of 12 days, for which resuscitation and therapeutic hypothermia had been necessary. He was treated with steroids. At the age of 18 months, he was suffering from proteinuria and low levels of vitB12, without anemia or other clinical manifestations. Oral supplementation of vitB12 (cyanocobalamin 1 mg daily) was started, and vitB12 deficiency was corrected.

We describe two siblings with IGS, who presented with vitamin B12 deficiency and proteinuria. The rare occurrence of this syndrome motivated us to provide an overview of all previously published cases and to discuss all aspects of IGS.

## Materials and methods

### Inclusion criteria

This is a retrospective study with no restriction on the publication date or language. The data were gathered using a *Pubmed* and *Google Scholar* search, using the keywords “Imerslund-Grasbeck” and/or “Imerslund Grasbeck”. All relevant articles, such as case reports, case series, and reviews, were reviewed. Additional cases were identified by consulting cited references of the identified articles. The included articles were selected based on title and abstract.

### Data collection

The following data were collected, if available: gender, consanguinity, origin, mutation analysis, age at onset and diagnosis, clinical manifestations at onset and diagnosis, laboratory examination (presence of pernicious anemia, vitB12 deficiency, proteinuria, Schilling tests, IF), examination of the renal system (including kidney biopsy and/or imaging, if applicable) and treatment. If the molecular analysis was not performed, information on IF analysis was included.

### Statistical analysis

We used descriptive statistics to summarize the data.

## Results

### Demographics

In total, 111 articles were included [2, 3, 5, 7–108]. 22 additional articles were not accessible. A total of 456 patients were included, of which 2 were described in this report.

### Origin

In 349/456 patients, ethnicity was documented. 22% were Turkish, 15% Finnish, 7% Norwegian, 6% Tunisian, 5% Bedouin and 6% were from the United States of America. All other origins occurred in less than 3%. Consanguinity of the parents was reported in 160/456 patients, of which 73% were consanguineous.

### Gender

Gender was reported in 278/456 patients of which 55% were male and 45% female.

### Age

The median age of onset of the symptoms was 2,3 years old (mean 4,2, SD ± 5,3 years, ranging from 0 months to 38 years old). The median age at diagnosis was 7,2 years old (mean 7,7, SD ± 7,9 years, range from 8 months to 48 years old).

### Gene and mutation analysis

The genetic analysis had been performed in 252/456 patients, 49% had a genetic variation in *AMN* and 51% in *CUBN*. In total, 119 different homozygous and compound heterozygous variants were detected, of which 34 were in *AMN* and 85 were in *CUBN*.

The most common variants in *AMN* were c.208-2 A > G (n = 78), c.14del6 (n = 22), c.1006 + 34_48del15bp (n = 18).

The most common *CUBN* mutations were c.3890 C > T (n = 36), c.434G > A (n = 22), c.2614_2615delGA (n = 14) and c.9053 A > C (n = 14).

### Diagnosis

In 52% of cases (235/456 patients), the diagnosis of IGS was confirmed by genetic analysis. In 22% (99/456 patients) the diagnosis was confirmed using a Schilling test and in 2% (9/456 patients), the diagnosis of the patient was suspected due to negative IF antibodies.

### Clinical manifestations

Information on clinical manifestations before diagnosis was provided in 325 of 456 patients (71%).

Gastrointestinal manifestations occurred in 103 patients (32%), including non-specific symptoms (diarrhea, vomiting, failure to thrive, n = 67, 51%), hepato(spleno)megaly (n = 38, 37%), and jaundice (n = 6, 6%). Mucosal lesions in the mouth, such as a sore tongue, recurrent oral ulcers and gingivitis were present in 54/325 patients (17%).

Respiratory symptoms were present in 9/325 (3%) patients, including recurrent respiratory infection, bronchopneumonia, and exertional dyspnea. Other symptoms included hypopigmented hair in 6 patients (1.8%) and local skin hyperpigmentation (n = 5, 1.5%). Other symptoms were present in only 1 or 2 patients (Additional file 1).

Neurological manifestations were observed in 52 patients (16%). Most frequently, patients showed lethargy (n = 13, 25%), ataxia (n = 10, 19%), and motor or sensory deficits (n = 7, 13%). More severe clinical manifestations were present in 4% of IGS patients, which included funicular myelosis (subacute combined degeneration of the cord, n = 5, 10%), seizures (n = 3, 6%), focal cortical atrophy of the left insular region on MRI (n = 2; 3.8%) and progressive dementia (n = 1, 2%). For the other neurological manifestations, we refer to Additional file 1.

In 247 patients, vitB12 levels were described, which were decreased in 94% of the patients. 89% of these patients had pernicious anemia.

In 239/456 patients, urinalysis was documented. Of these patients, 212 (89%) had proteinuria. Only in Finland, the fraction of patients with proteinuria was lower than in other countries (20% had proteinuria, 18% had no proteinuria, 62% had no urinalysis).

### Comorbidities

Of the 325 patients with reported clinical circumstances, 48 (15%) had associated comorbidities in addition to IGS (Table 2). Abnormalities in the urogenital tract were most often observed, which included anatomic nephrogenic or urinary tract abnormalities (n = 10, 3%) and vesicoureteral reflux or IgA nephropathy (both n = 1, 0.3%).

**Table 2:** Comorbidities in IGS patients, expressed in number of patients *n* and percentage % (of the total amount of patients in which clinical characteristics were described in the article; 325 patients)

*Comorbidity*	*n*	%
**Urinary tract**	18	5.5
Anatomical urinary tract anomalies	10	3
Alport syndrome	1	0.3
Focal segmental glomerulosclerosis	4	1.2
IgA nephropathy grade I	1	0.3
Membranous glomerulonephritis	1	0.3
Vesico-urethral reflux grade II	1	0.3
**Haematological**	11	3.4
Alpha thalassemia	2	0.6
Alpha thalassemia trait	2	0.6
Bèta thalassemia	2	0.6
Bèta thalassemia trait	1	0.3
Defective neutrophil function	3	0.9
Sickle anemia	1	0.3
**Immunological/endocrine disorders**	11	3.4
Congenital adrenal hyperplasia	1	0.3
Congenital hypothyroidy	1	0.3
Eosinophilic enterocolitis	2	0.6
Fanconi anemia	1	0.3
Gluten intolerance	1	0.3
IgA deficiency	3	0.9
Type I diabetes	2	0.6
**Ophthalmological abnormalities**	3	0.9
Cataract	1	0.3
Partial atrophy of optic nerves	1	0.3
Congenital retinal atrophy	1	0.3
**Other**	10	3
Gastroschisis	1	0.3
Prematurity	3	0.9
Pulmonary tuberculosis	2	0.6
Rectal polyp	1	0.3
Right-side heart failure	1	0.3
Trisomy 21	1	0.3
Thrombotic microangiopathy	1	0.3

In 19 patients, a kidney biopsy was performed. Most patients exhibited glomerular abnormalities (n = 17, 5.2% of 325 patients) including 4 cases of focal segmental glomerulosclerosis. Some patients exhibited tubular abnormalities (n = 2, 0.6% of 325 patients). No deterioration of kidney function has been reported in any IGS patients, to the authors’ knowledge.

The other most common comorbidities were thalassemia (n = 7, 2.2%), IgA deficiency and defective neutrophil function (both n = 3, 0.9%). Other comorbidities occurred in only 1 or 2 patients.

### Therapy

The method of vitB12 therapy was described in 240/456 cases. VitB12 was administered intramuscularly in 88%, parenteral (either intravenous or intramuscular, but not further specified) in 2%, and orally in 9% (including in our patients).

## Discussion

### Vitamin B12 physiology

VitB12 is a water-soluble vitamin most commonly found in animal products. It serves as a cofactor for enzymes involved in metabolism. Upon entry into the stomach, vitB12 is released from dietary proteins and bound to haptocorrin. The complex consisting of vitB12 and haptocorrin is broken down in the intestine and a complex consisting of vitB12 and IF is formed [109]. In the terminal ileum, it binds to the cubam receptor located on the ileal apical brush border, and the cubam complex is internalized by endocytosis. In the endosome, the IF-vitB12 complex is released from the cubam receptor. VitB12 is released from IF in the lysosome and exported to blood [109–111]. In the blood, it is transported bound by transcobalamin II, which also enables vitB12 to enter target cells. In the target cells, vitB12 is released into the lysosome, enabling it to serve as a cofactor in metabolic processes [109, 111]. VitB12 deficiency can lead to the deficiency of the cofactors adenosylcobalamin and methylcobalamin. Adenosylcobalamin is a cofactor of the enzyme methylmalonyl-coenzyme A mutase which enables the degradation of methylmalonyl-CoA into succinyl-CoA, which is a Kreb cycle intermediate. This is the final step in the degradation of the aminoacids isoleucine/valine/threonine and odd-chain fatty acids [112]. Methylcobalamin is a cofactor of methionine synthase, which is responsible for converting homocysteine into methionine. Methionine is then converted to S-adenosylmethionine, which is essential for the methylation of several compounds such as creatine, epinephrine, DNA, RNA and proteins [113].

### Pathophysiology

IGS is an autosomal recessive disorder caused by pathogenic variants in either *CUBN* or *AMN*, which encode the 2 subcomplexes of the cubam receptor: cubilin and amnionless. Cubilin is a large protein that can bind several ligands and is responsible for the uptake of vitB12, but also for renal tubular reabsorption of other proteins after glomerular ultrafiltration [106]. As it has no transmembrane region, it is bound to the transmembrane protein amnionless, which also mediates internalization of the IF-vitB12-cubam complex [83, 106].

It has been demonstrated that the binding of amnionless to cubilin occurs very early in its biogenesis. After that, cubilin undergoes a posttranslational modification in the endoplasmatic reticulum (ER), after which the complex is packed in the vesicles of the Golgi apparatus and transported to the plasma membrane [114]. Studies have shown that cubilin and amnionless are mutually dependent on each other and that none of the two proteins can reach the plasma membrane if the other is deficient [106]. Udagawa et al. [106] described several mechanisms underlying impairment in renal and intestinal absorption in IGS patients. Variants in either *CUBN* or *AMN* caused defects in intracellular trafficking, accumulation in the ER and therefore decreased membrane expression in vitro. This was confirmed in vivo by a kidney biopsy of an IGS patient with a *CUBN* mutation. There was an expression of cubilin and amnionless in the ER of proximal tubular cells but not in the brush border of proximal tubular cells, where cubilin and amnionless should be present. Furthermore, *AMN* variants result in defective binding of cubilin to amnionless, which was not observed in *CUBN* variants. Finally, both *AMN* and *CUBN* variants cause disrupted glycosylation of cubilin. *N*-glycosylation is a posttranslational modification process that regulates protein folding, protein stability, protein complex formation and intracellular trafficking. Specific *N-*glycosylation in cubilin domains is be essential for cubam complex maturation and surface localization [106].

### Clinical manifestations

Patients may develop symptoms once fetal hepatic vitB12 is exhausted, generally between the ages of 1 and 5 years [112]. Patients may present with non-specific symptoms such as failure to thrive, general weakness, and recurrent gastro-intestinal and/or respiratory infections. In addition to non-specific symptoms, we observed a relatively high amount of oral mucosal lesions in 17% of the patients. We did not observe any relations between these non-specific symptoms and genotype.

Neurocognitive symptoms have been attributed to several pathophysiological mechanisms, including decreased myelinization as a result of impaired methylation due to vitB12 shortage and the direct toxic effects of homocysteine and methylmalonic acid [109]. Neurological symptoms are reported to be generally mild and non-specific such as developmental delay, learning difficulties, and psychological symptoms due to vitB12 deficiency [1, 5]. In our review, we observed a surprisingly high incidence of moderate (ataxia in 4% of IGS patients) and even severe neurological manifestations including patients with convulsions, degeneration of the spinal cord, focal cortical brain atrophy, and progressive dementia (3.5% of IGS patients) [48, 71, 72, 74]. The numbers of these severe neurological manifestations were, however, too small to draw conclusions about phenotype-genotype correlations.

### Proteinuria and renal manifestations

Proteins that are found in the urine are predominantly middle molecular weights proteins with a high fraction of albumin (61–100%) [2, 27]. Aminoaciduria has been described by some authors [2, 27, 100, 115]. Only very small amounts of high molecular weight proteins, such as IgG, were found [27].

The pathophysiology of proteinuria has not been fully elucidated. The proteinuria is not of the classical glomerular or tubular type, but probably results from the lack of cubilin function that is needed for tubular reabsorption of some, but not all, urinary proteins [52]. Both cubilin and amnionless are highly expressed in the proximal tubules of the kidney. In the proximal tubule, but not in the intestine, they interact with the endocytic receptor megalin, which leads to the internalization of the cubilin-amnionless complex (cubam) and thus enables the reabsorption of filtered plasma proteins [83, 106]. In the case of a mutation in *CUBN* or *AMN*, retention of cubilin and amnionless in the ER of proximal tubular cells can occur, leading to the absence of cubam on the cell surface and deficient reabsorption of albumin in the proximal tubule, resulting in proteinuria [106]. There have been some observations of tubular abnormalities in patients who had a kidney biopsy, including tubular atrophy and the presence of giant mitochondria in epithelial cells of the proximal tubule [25, 106].

There is, however, also some evidence of a glomerular origin of proteinuria and several authors described glomerular abnormalities in patients who had a kidney biopsy. This included pathological changes in podocytes (cubilin is also expressed in podocytes), which may contribute to proteinuria [3, 5, 10, 22, 25, 27, 38, 68, 102, 104]. Evidence of focal segmental glomerulosclerosis with glomerular mesangial cell proliferation, interstitial fibrosis, and structural abnormalities of podocytes including effacement of foot processes and podocyte microvillation, has also been observed [10]. Other authors have found similar abnormalities of slight chronic glomerulopathy or glomerulonephritis [3, 10, 27, 104].

The pathophysiology of these renal manifestations remains poorly understood. The involvement of megalin, has been suggested in the pathophysiology of renal abnormalities [106]. Megalin can bind albumin, while cubilin is thought to have a higher binding affinity to albumin as compared to megalin. Some authors hypothesized that in the absence of normal cubilin, a higher amount of albumin is bound to megalin, promoting a pathological cascade resulting in podocyte apoptosis via the PI-3 K/PKB pathway, resulting in changes in glomerular basement membrane abnormalities and focal segmental glomerulosclerosis [10].

Kidney biopsy has been performed only rarely, because in the last years, it has become clear that although proteinuria persists, renal abnormalities do not progress in IGS patients that are treated with vitB12 ^1^. The reason for this observation is unknown, but it has been suggested that unlike proteinuria, there may be small changes in kidney ultrastructure that respond to vitB12 supplementation [112].

In our series, 89% of IGS patients had proteinuria. Smaller numbers have been reported earlier [1, but these were small series within one region. There are several reasons for overestimation.

Firstly, urinanalysis was documented in 232 patients, but clinical features were documented in 325 patients. It may be possible that 89% proteinuria is an overestimation and that the number of patients with proteinuria may be closer to 62% (203 patients with proteinuria among 325 patients in which clinical characteristics were described). Secondly, given that only 92 of those patients had genetically confirmed IGS (40%), some of the patients may strictly not be IGS. However, due to the combination of vitB12 deficiency and proteinuria, IGS is very likely. Thirdly, there may be an overestimation because patients without proteinuria may not all be diagnosed. A combination of vitB12 deficiency and proteinuria prompts further diagnostic testing for IGS. An absence of proteinuria, however, may not lead to diagnostic testing for IGS. Fourthly, we observed that Finnish patients less frequently exhibited proteinuria. As Finland is one of the countries in which IGS is more common as compared to other countries, there may be a large number of unreported cases.

Tanner et al. [5] previously hypothesized that proteinuria may only be observed in patients harboring *AMN* or *CUBN* variants that encode the cubilin-amnionless interaction domain, but not in patients with variants in *CUBN* in the IF-binding site [5]. However, we also observed patients with *AMN* variants without proteinuria.

Urinary tract abnormalities were observed in 10 patients (3.1% of IGS patients), of which 6 patients had genetic testing. In all cases, *AMN* variants were identified. *CUBN* variants were not identified in this specific cohort. Urinary tract abnormalities have indeed, been previously associated with *AMN*-related IGS, reflecting the role of AMN during embryonic development [1, 116]. The absence of *CUBN* variants in patients with urinary tract anomalies suggests a difference in the phenotypic spectrum with potential genotype-phenotype correlation. However, the numbers are too small to draw definitive conclusions. Also, congenital anomalies of the kidney and urinary tract are not rare. They represent approximately 20–30% of all anomalies identified in the prenatal period [117].

### Diagnosis

As described above, the observation of macrocytic anemia, vitB12 deficiency, and permanent proteinuria is highly suggestive of IGS. Because we observed that 92% of patients with IGS had proteinuria, we recommend health care providers to examine the urine in any child with a vitB12 deficiency.

Other clinical diagnostic findings may include thrombocytopenia, moderate leukopenia and neutropenia, and high levels of homocysteine and methylmalonic acid in serum or urine [2, 3, 5].

IGS is diagnosed by detecting vitB12 deficiency and the molecular analysis of the *CUBN* and *AMN* genes to confirm IGS, and the *CBLIF* [Genbank NG_008120] gene to exclude IF-deficiency. If a molecular analysis is unavailable, serum vitB12, folate, methylmalonyl acid (accumulates in vitB12 deficiency but not in folate deficiency), homocysteine, and a myelogram (to rule out malignant conditions) may be helpful [1]. Antibodies against IF can be investigated to exclude deficiency of IF as a cause of megaloblastic anemia [5]. IF assays in the gastric fluid may be used to differentiate between IGS and IF deficiency [1].

In addition, a test to demonstrate that vitB12 is poorly absorbed, such as the Schilling test, a radiolabeled vitB12 absorption test to confirm vitB12 malabsorption [1, has been used. However, this test does not distinguish between IGS and congenital IF deficiency (absence or production of inert IF) [27]. In the two-stage Schilling test, IF was added to one of the steps, which resulted in the correction of IF deficiency, but not of IGS. Another limitation of the Schilling test is that vitB12 deficiency may affect the enterocyte function, leading to secondary malabsorption and an abnormal Schilling test [5]. Currently, the Schilling test is not available in most countries due to the difficulty to obtain labeled cobalamine. Other tests using, for example cyano-vitB12 to measure the fraction of transcobalamin bound to cyano-vitB12, are proposed by previous authors, who however, also expressed concerns about its sensitivity [5]. No replacement test has been validated. A therapeutic test with vitB12 or folate can be useful.

### Mutations

In 1999, *CUBN* mutations were first described in a group of Finnish patients with vitB12 deficiency and proteinuria, followed by *AMN* mutations observed in Norwegian and Jewish patients [51, 81]. Because *CUBN* and *AMN* were not recognized until after 1999 as the genes responsible for IGS, many of the previously published cases of IGS were found not to be due to IGS but rather to defects in IF [1]. Tanner et al. [5] carried out genetic screening in 154 cases of suspected inherited vitB12 malabsorption. Of these patients, 64% of patients had either a *CUBN* or *AMN* mutation, 18% of patients had a *CBLIF* mutation, and 18% had no mutation that could be identified. The authors suggested several other candidate genes based on their role in vitB12 transport, but none of these mutations could be confirmed in their patients [5].

We found 119 different mutations in either *CUBN* or *AMN* genes. In 48% of cases that have been classified as IGS, genetic testing has not been performed. In 24% of these patients, other diagnostic tests were carried out suggesting IGS, including in 22% a Schilling test and in 2% a test to rule out an IF deficiency.

Most of the patients have proteinuria, and therefore probably IGS. But some of the patients may have another cause of vitB12 deficiency, as suggested by Tanner et al. [5]. However, due to the limitations described in the previous section, these cases should be (if possible) reassessed using molecular analysis to either confirm or reject the diagnosis of IGS.

### Comorbidity

In 15% of IGS patients, other diseases in addition to IGS were present, such as congenital adrenal hyperplasia in patient 2. As previously mentioned, patients with IGS probably have a high rate of comorbidity due to the high rate of consanguinity [1]. Urinary tract abnormalities are observed relatively frequently, the pathophysiology of which is described in more detail earlier in this report.

### Treatment

Initially, vitB12 deficiency in IGS patients was corrected with monthly intramuscular injections of vitB12. One would expect a lack of effect of oral vitB12 since IGS is caused by malabsorption of vitB12 ^2^. However, as with our patient, effective oral treatment with vitB12 has been previously described. Oral treatment is based on the assumption that if the dose is high enough, a small but still sufficient part is absorbed [118]. Because approximately 1% of oral vitB12 is absorbed by passive diffusion in its free form, high doses can effectively treat vitB12 deficiency, also in patients with IGS [119]. VitB12 needs to be administered throughout life in IGS [1].

### Prognosis

IGS patients treated with vitB12 are clinically and hematologically normal. The proteinuria persists but does not increase in severity The kidney function does not deteriorate [1, 27]. However, to the authors’ knowledge, the long-term studies only described patients up to the age of 46 years [27].

### Limitations

Firstly, the clinical details of the IGS patients described above are limited. Secondly, due to the lack of genetic testing in 48% of the patients included in this study, the diagnosis of IGS cannot be completely certain. Thirdly, particularly in relatively high-prevalence countries such as Norway and Finland, many cases may not have been reported. Finally, IGS may be grossly under-diagnosed as most patients present with non-specific symptoms. While a combination of vitB12 deficiency and proteinuria leads to additional diagnostic tests to diagnose IGS, in the absence of proteinuria, IGS may not be recognized. We also observed a high rate of oral mucosal lesions and neurological symptoms such as ataxia, all of which can lead to further diagnostic testing. Therefore, we expect that IGS will be significantly underdiagnosed and that we observed an overestimation of some clinical manifestations.

## Conclusions

IGS is a rare autosomal recessive disorder characterized by vitB12 deficiency and in most patients, proteinuria. We reviewed the literature on IGS and, for the first time, all previously published cases of IGS. In addition, we describe two new patients. Most patients present with non-specific symptoms. If untreated, patients may develop severe (neurological) manifestations. Patients may be treated with oral vitB12 supplementations but may need higher doses. Because 92% of patients with IGS had proteinuria, we advise health-care providers to examine the urine of any child with vitB12 deficiency.

### Electronic supplementary material

Below is the link to the electronic supplementary material.

Supplementary Material 1

Supplementary Material 2

## Data Availability

All data generated or analyzed during this study are included in this published article [and its supplementary information files].

## Abbreviations

*AMN*: Amnionless/amnion-associated transmembrane protein
CBLIF: Cobalamin binding intrinsic factor
*CUBN*: Cubilin
*CYP21A2*: cytochrome P450 family 21 subfamily A member 2
ER: Endoplasmatic reticulum
IF: Intrinsic factor
IGS: Imerslund-Grasbeck syndrome
LDH: Lactate dehydrogenase
VitB12: Vitamin B12
