# Supplementary material for: Imerslund-Gräsbeck syndrome: a comprehensive review of reported cases
Source: Orphanet J Rare Dis. 2023 Sep 14;18:291. doi: 10.1186/s13023-023-02889-x (PMC10500774; doi:10.1186/s13023-023-02889-x)
Supplement: Supplementary file 1 — Supplementary Material 1 [file 13023_2023_2889_MOESM1_ESM.docx]

January 17, 2023

Prof. Francesc Palau, Editor-in-Chief

Orphanet Journal of Rare Diseases

Dear prof. Palau,

Please find enclosed the original copy of our manuscript entitled "**Imerslund-Gräsbeck syndrome: a comprehensive review of reported cases**", by Sandra D.K. Kingma, Julie Neven, An Bael, Marije E.C. Meuwissen and Machiel van den Akker, for consideration of publication in Orphanet Journal of Rare Diseases.

Imerslund-Gräsbeck syndrome is a rare autosomal recessive disorder characterized by vitamin B12 malabsorption and often proteinuria. If untreated, patients may develop sometimes severe neurological manifestations.

After the diagnosis of two siblings with Imerslund-Gräsbeck syndrome in our center, we reviewed the literature and all previously published cases of Imerslund-Gräsbeck syndrome. This manuscipt provides a complete overview of Imerslund-Gräsbeck syndrome and we hypothesize that Imerslund-Gräsbeck syndrome may be severely underdiagnosed.

We descibe a surprisingly high incidence of often severe neurological symptoms. To prevent severe neurological complications, early diagnosis is essential and awareness of Imerslund-Gräsbeck syndrome is an important first step.

By submitting the manuscript to the Journal, the authors understand that the material presented in this paper has not been published before nor has it been submitted for publication to another scientific journal or being considered for publication elsewhere. I attest that this work has been approved by all co-authors. The authors also understand that should the submitted material be accepted for publication in the journal, they will automatically transfer the copyright to the publisher.

We feel that our manuscript is a valuable addition and update of the excellent review of Gräsbeck that was published in OJRD in 2006.

I hope that the reviewing process finds the manuscript acceptable for publication.

Yours sincerely,

Dr. Sandra Kingma

Dr. Machiel van den Akker
